# Infliximab-Induced Lupus in a Patient With Psoriatic Arthritis Who Presented With Cardiac Tamponade: A Case Report

**DOI:** 10.7759/cureus.36424

**Published:** 2023-03-20

**Authors:** Liyan Obeidat, Linda Albusoul, Mohamed Maki, Hanan Ibrahim, Sachin Parikh

**Affiliations:** 1 Internal Medicine, Henry Ford Health System, Detroit, USA; 2 Rheumatology, Henry Ford Health System, Detroit, USA; 3 Cardiology, Henry Ford Health System, Detroit, USA

**Keywords:** psoriatic arthritis, anti-tumor necrosis factor-α, infliximab induced lupus, acute pericarditis, drug induced lupus, cardiac tamponade

## Abstract

Psoriatic arthritis (PsA) is a chronic, immune-mediated inflammatory condition, and the proinflammatory cytokine tumor necrosis factor-α (TNF-α) plays a major pathogenic role in the development and progression of PsA. Anti-TNF-α therapies, such as the monoclonal antibody infliximab, are used to treat patients whose PsA has not responded favorably to conventional anti-rheumatic drugs. However, exposure to anti-TNF-α therapeutics can lead to drug-induced lupus erythematosus (DILE), which may rarely be accompanied by cardiac manifestations. Here, we describe a rare case of drug-induced lupus erythematosus secondary to infliximab therapy for PsA and psoriasis in a patient who presented with life-threatening acute pericarditis and cardiac tamponade. Newly developed skin rashes, newly elevated autoimmune indicators, and punch biopsy results indicating subacute cutaneous lupus collectively supported a DILE diagnosis within the context of infliximab use. Pericardiocentesis, colchicine, and corticosteroids alleviated symptoms, and infliximab was replaced with alternate therapy. This case highlights the importance of early recognition of the possible serious and uncommon adverse reactions from infliximab therapy. Prompt initiation of appropriate treatment and discontinuation of the offending agent are critical in cases of drug-induced lupus erythematosus, particularly when rare cardiac complications occur.

## Introduction

Psoriatic arthritis (PsA) is an autoimmune inflammatory arthritis that affects the peripheral joints, axial skeleton, skin, and entheses. Due to their effectiveness in maintaining remission, anti-tumor necrosis factor-α (TNF-α) agents, such as infliximab, were introduced for the treatment of patients with psoriasis and PsA who had failed to respond to traditional therapies. Drug-induced lupus erythematosus (DILE) is a clinical syndrome that can develop following exposure to many medications, including anti-TNF-α agents, as a result of an autoimmune response and autoantibody formation. It presents with symptoms similar to those of systemic lupus erythematosus (SLE). Common manifestations of DILE include a cutaneous rash, inflammatory arthritis, leukopenia, thrombocytopenia, hemolytic anemia, and other systemic manifestations [1]. Pleural or pericardial inflammation, pericardial effusion, and cardiac tamponade only rarely occur. Our case describes a rare presentation of DILE with life-threatening cardiac tamponade, supported by serological evidence and tissue biopsy findings.

## Case presentation

A 46-year-old woman presented to the emergency department with shortness of breath and severe positional pleuritic chest pain, which worsened with deep breathing and with lying down. The patient had a medical history of psoriasis and PsA since childhood and was currently receiving treatment with weekly methotrexate (20 mg) and monthly infusions of infliximab (7 mg/kg). She reported not having any fever, chills, cough, abdominal pain, diarrhea, night sweats, recent unintentional weight loss, or aggravation of her chronic joint pain. She mentioned that she had just developed a rash on her neck, which she attributed to her history of psoriasis.

At presentation, she had normal blood pressure but significant tachycardia, with a heart rate of 130 beats per minute. Her oxygen saturation was 92% on room air. Physical examination showed jugular venous distension and decreased heart sounds on auscultation. A skin examination showed psoriatic lesions on her lower extremities and psoriasiform lesions in the upper neck area that were new. There was no joint tenderness, erythema, swelling, warmth, deformities, dactylitis, or tenderness at the entheseal sites noted on the musculoskeletal examination.

Initial laboratory results revealed leukocytosis with neutrophilic predominance, mild anemia, high-sensitivity troponin within the normal range, slightly elevated brain natriuretic peptide (BNP), normal ferritin levels, elevated C-reactive protein (CRP), and an elevated erythrocyte sedimentation rate (ESR) (Table 1).

**Table 1:** Laboratory data at the initial presentation.

Laboratory tests	Reference range	Results
Hemoglobin, g/dL	12.0–15.0	11.5
White cell count, K/µL	3.8–10.6	16.3
Neutrophil %	%	73
Lymphocyte %	%	14
Absolute neutrophilic count, K/µL	1.80–7.70	11.9
Absolute lymphocytic count, K/µL	1.10–4.00	2.28
Absolute monocytic count, K/µL	0.00–0.80	1.79
Platelets, K/µL	150–450	439
High sensitivity troponin, ng/L	<4	4
Brain natriuretic peptide, pg/mL	<50	87
Ferritin, ng/mL	11–307	124
C-reactive protein, mg/dL	<0.5	19
Erythrocyte sedimentation rate, mm/hr	<20	81

**Figure 1:**
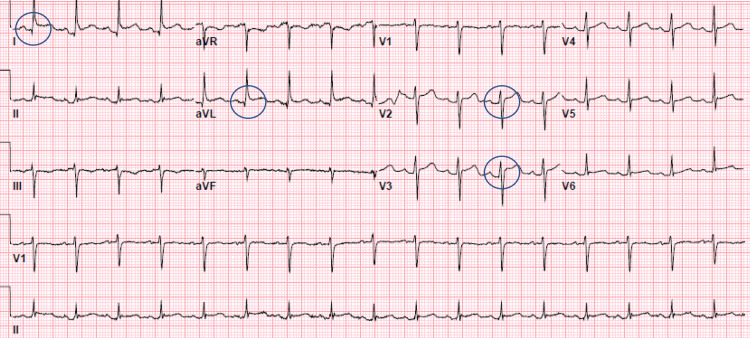
Electrocardiogram demonstrating sinus tachycardia at a rate of 103 bpm, diffuse ST-segment elevation, and PR segment depression primarily in leads 1, avL, V2, and V3 (circles).

There was no acute cardiopulmonary process on the chest X-ray. The electrocardiogram demonstrated sinus tachycardia, diffuse ST-segment elevation, and PR-segment depression consistent with acute pericarditis (Figure 1).

**Figure 2:**
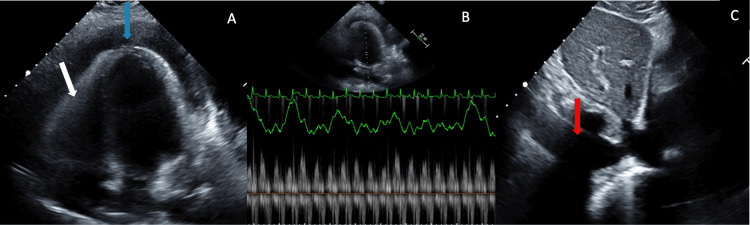
Transthoracic echocardiogram findings. (A) Apical window showing circumferential large pericardial effusion (blue arrow) with evidence of diastolic right ventricular wall collapse (white arrow); (B) excessive respiratory variation in the mitral valve spectral doppler velocities; (C) dilated and nonreactive inferior vena cava (red arrow).

An echocardiogram showed a circumferentially large pericardial effusion around the entire heart; the largest effusion was noted in the apical window, with evidence of diastolic right ventricular wall collapse, excessive respiratory variation in the mitral valve spectral doppler velocities (more than 25%), as well as a dilated, nonreactive inferior vena cava, all of which were suggestive of cardiac tamponade physiology (Figure 2).

She was taken for pericardiocentesis, and 270 ml of serous fluid was drained. Fluid analysis showed a total nucleated cell count of 5822/cu mm, with 64% neutrophils, 19% lymphocytes, 16% monocytes, and 1% basophils. Fluid pH was 7.35, the protein was 6.5 g/dL, and the glucose was 45 mg/dL. Fluid bacterial, fungal, and acid-fast bacilli cultures were negative, and fluid cytology was negative for malignant cells.

The rheumatology team was consulted, and further workup showed positive antinuclear antibodies (ANA), normal complement factor-3 (C3), normal complement factor-4 (C4), negative anti-Sjögren's syndrome type A (SSA) antibody, negative anti-Sjögren's syndrome type B (SSB) antibody, and negative anti-Smith antibody. New positive rheumatoid factor (RF), anti-ribonucleic protein (RNP) antibody, anti-histone antibody, and anti-double stranded DNA (dsDNA) antibody, all of which were previously checked at the time of diagnosis and were negative (Table 2).

**Table 2:** Rheumatology workup throughout the course of hospital stay. IFA: indirect immunofluorescence assay.

Test	Value	Reference range
RF	27	<14 IU/mL
ANA	Positive	Negative
Anti-SSA/Ro antibody	<0.2	<1.0 Elisa units
Anti-SSB/La antibody	<0.2	<1.0 Elisa units
Anti-RNP antibody	1.1	<1.0 Elisa units
Anti-Smith antibody	4	<20 units
Anti-histone antibody	3.1	<1.0 units
Anti-dsDNA antibody, IFA	Positive, 1:160	Negative, <1:10 titer
C3	150	90–230 mg/dL
C4	19	10–51 mg/dL

**Figure 3:**
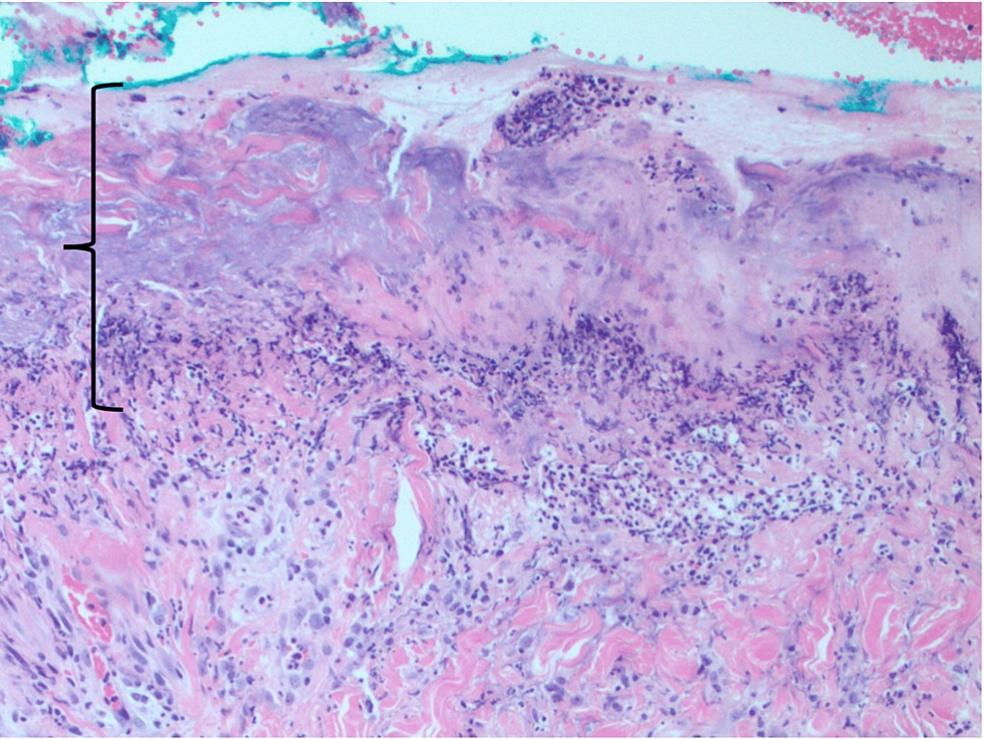
Skin biopsy findings showing ulceration, likely secondary to excoriation, with diffuse and papillary dermal neutrophilic infiltrates (marked area), concerning for bullous lupus erythematosus in this clinical setting.

She was evaluated by a dermatologist for the newly discovered rash, and a punch biopsy was obtained and revealed results that were consistent with subacute cutaneous lupus (Figure 3).

She was started on colchicine 0.6 mg twice per day and low-dose prednisone tapered over time for both pericarditis and joint pain. She had significant improvement in her symptoms with treatment and no recurrence of pericardial effusion.

After reviewing the patient’s case with the cardiology, rheumatology, and dermatology teams, it was concluded that her presentation was consistent with DILE related to infliximab use. Her presentation with acute pericarditis and cardiac tamponade, alongside a new skin rash and several positive serology results, all supported this conclusion. As a result, infliximab was discontinued, and she was started on guselkumab, a monoclonal antibody against interleukin-23. The anti-dsDNA antibody was checked during a follow-up appointment with rheumatology one month after discharge, and the result was negative.

## Discussion

DILE is a clinical syndrome that shares characteristics with SLE and can arise as a result of exposure to medications that have the potential to trigger an autoimmune reaction, such as procainamide, hydralazine, anti-TNF treatments, and many others [2-4]. The demographics of DILE vary greatly depending on the individual using the medication, and they tend to be more prevalent in females, older persons, and white populations [5,6]. According to numerous reviews, the clinical and serologic pattern of DILE varies substantially across agents, and there is no single pattern seen in all of them. Seroconversion and the development of autoantibodies with the use of anti-TNF can happen without a clinically significant disease in the majority of cases. Although rare, exposure to such medications can induce lupus, as demonstrated in our case [1].

Human TNF has a variety of biological effects, including the production of interleukins and cytokines that promote inflammation, the stimulation of leukocyte activation, and the production of acute phase reactants and tissue-degrading enzymes [7]. Infliximab is a chimeric monoclonal antibody that binds to human TNF-α, thereby inhibiting its endogenous activity. It is frequently used to treat a variety of rheumatologic conditions, including PsA, ankylosing spondylitis, and rheumatoid arthritis, as well as inflammatory bowel disease, in which high tissue TNF levels have been linked to tissue destruction [7-10].

Although the exact mechanism by which anti-TNF-α medication induces DILE is still unknown, several theories have been put forth, all of which involve the development of autoantibodies. One theory suggests that the T helper type 1 response is suppressed by anti-TNF drugs, which results in a shift toward the T helper type 2 predominant response and the production of autoantibodies. Another source stated that because anti-TNF drugs increase the risk of infections, they can result in lymphocyte activation and the formation of antibodies [9]. There were also some genetic risk factors that have been identified [1].

Compared to other drugs that cause DILE, infliximab carries a very low risk of approximately 0.1% [11]. The presentation of DILE associated with the use of infliximab and other TNF-α inhibitors is typically mild with a lower incidence of life-threatening organ dysfunction when compared to idiopathic SLE [4]. Some rare clinical manifestations may develop, such as serositis, pleural effusions, pneumonitis, neuritis, and immune complex-mediated glomerulonephritis [7]. The occurrence of acute pericarditis with cardiac tamponade, which is characterized by pericardial inflammation and fluid buildup in the pericardial sac, raising intracardiac pressure and lowering cardiac output, is a rare and potentially fatal presentation. It necessitates proper emergent management with pericardiocentesis or a pericardial window. Direct cardiac toxicity or a type 3 hypersensitivity immune-complex reaction that manifests like serum sickness are two other pathways described in the development of infliximab-induced pericarditis [2,12].

High ANA and anti-dsDNA antibody titers were present in the majority of DILE cases related to anti-TNF-α use. It has been demonstrated that these medications may induce new-onset autoantibody formation, which is rare enough to cause a clinical syndrome [13]. In contrast to findings with other medications causing DILE, anti-histone antibodies are usually absent [4,10]. Our patient was found to have new positive results of anti-dsDNA as well as anti-histone antibodies coinciding with the onset of cardiac tamponade, making the diagnosis of DILE most favorable. It is important to note that the majority of patients who acquire ANA or anti-dsDNA antibodies do not have the clinical syndrome of DILE, and those antibodies should only be interpreted in the right clinical setting to avoid withholding effective therapies [10].

According to our literature search, the first instance of cardiac tamponade as a presenting symptom of DILE related to infliximab use was published in 2014, and subsequent cases were reported in patients being treated for inflammatory bowel disease and rheumatoid arthritis [4]. To the best of our knowledge, this is the first case of cardiac tamponade related to infliximab use in PsA. Our patient's presentation included both typical and atypical DILE manifestations, coinciding with the presence of positive serological evidence, along with a tissue biopsy that supported the diagnosis.

## Conclusions

Rarely, pericarditis and cardiac tamponade may be seen in patients who develop infliximab-induced lupus. As suggested by this case, when no other cause is immediately evident, DILE should be considered in the differential diagnosis of patients with rheumatic conditions who develop cardiac tamponade within the context of infliximab or other anti-TNF therapies, particularly within the setting of a newly developed rash and elevated autoimmune markers. Pericardiocentesis, initiation of colchicine, steroid therapy, and most importantly discontinuation of infliximab should lead to timely remission of cardiac symptoms and prevention of recurrence.
